# Interleukin-27–producing cells in gram-negative neonatal sepsis display diverse phenotypes and functions in the liver

**DOI:** 10.1093/immhor/vlaf026

**Published:** 2025-07-18

**Authors:** Jordan K Vance, Nathalie Lailler, Ashley M Divens, Jessica M Povroznik, Madhavi Annamanedi, Kathleen M Brundage, Cory M Robinson

**Affiliations:** Department of Microbiology, Immunology, and Cell Biology, West Virginia University School of Medicine, Morgantown, WV, United States; Rancho Biosciences, San Diego, CA, United States; Department of Microbiology, Immunology, and Cell Biology, West Virginia University School of Medicine, Morgantown, WV, United States; Department of Microbiology, Immunology, and Cell Biology, West Virginia University School of Medicine, Morgantown, WV, United States; Department of Microbiology, Immunology, and Cell Biology, West Virginia University School of Medicine, Morgantown, WV, United States; Department of Microbiology, Immunology, and Cell Biology, West Virginia University School of Medicine, Morgantown, WV, United States; Department of Microbiology, Immunology, and Cell Biology, West Virginia University School of Medicine, Morgantown, WV, United States; Vaccine Development Center, West Virginia University Health Sciences Center, Morgantown, WV, United States

**Keywords:** interleukin-27, macrophages, neonatal, sepsis, transcriptome

## Abstract

Neonates have increased vulnerability to life-threatening infections due to the distinct immune landscape. Interleukin (IL)-27 is a key component of this immune profile that we have previously shown to be elevated in both newborn humans and mice. IL-27 continues to increase in the serum and tissues consistent with poor outcomes during gram-negative neonatal bacterial sepsis. Presently, we dissected the IL-27 producer profile at a single-cell level using IL-27p28eGFP reporter mice in our previously established model of neonatal sepsis with luciferase-expressing K1-encapsulated *Escherichia coli*. Whole animal imaging regionally highlighted the spleen, liver, and lungs as key infection sites by bacterial luminescence. Flow cytometry showed that IL-27 producers increased significantly in the liver with infection and were predominantly F4/80^+^ and CD11b^+^ with subpopulations that emerged expressing additional markers. This information paired with single-cell RNA sequencing further identified the most robust populations as monocytes, monocyte-derived cells, and Kupffer cells followed by smaller populations of dendritic cells and neutrophils. The transcriptome demonstrated a diverse range of functionality amongst populations that included differential expression of genes implicated in bactericidal, metabolic, and inflammatory changes. Collectively, the transcriptome of IL-27 producers from the livers of infected animals suggests an uncoordinated mix of inflammatory and suppressive activity that may contribute to immune dysregulation characteristic of sepsis. Together, this work provides previously undescribed insight into the details of IL-27 producers during early-life infection. This further provides essential information needed to support IL-27 as a therapeutic target for neonatal bacterial sepsis.

## Introduction

There are distinct contrasts in the early-life immune response to what is observed in the adult immune system. A consequence of this immunological profile, generally considered to be more suppressive and immune tolerant in newborns, is a heightened susceptibility to life-threatening infections. Globally, there were over 2 million neonatal deaths in 2018 and 2022, which constituted almost half of all deaths in children under 5 yr old.^1^^,^^2^ A significant proportion of this mortality is attributed to infectious disease. Neonatal sepsis specifically affects 2 newborns out of every 100 live births each year, with the overall global incidence most likely underreported and increasing annually.^3^^,^^4^

Some of the unique characteristics of the neonatal immune system include decreased expression of T helper 1 (Th1)–associated cytokines such as interleukin (IL)-12, interferon-γ, and tumor necrosis factor-α along with increased expression of the Th17-associated cytokines IL-6 and IL-23 when compared with adults.^5–7^ Together, this promotes a Th2-biased, generally less inflammatory humoral immune response. This immunological phenotype is more permissive of potential pathogens, such as *Escherichia coli* and Group B streptococci, which are commonly responsible for neonatal bacterial sepsis.^8^ While Group B streptococci are the leading cause of early-onset sepsis overall, *E. coli* is responsible for the majority of deaths and the leading cause when preterm and low-birth-weight babies are considered independently.^9^^,^^10^

To add to the current understanding of the early-life immune landscape, our laboratory has identified increased levels of IL-27 produced by neonatal human cells.^11^^,^^12^ These findings are consistent in mice, in which we continue to investigate the impact of early-life IL-27 levels on the immune system.^11^^,^^13^ IL-27 is a pleiotropic immune-suppressive cytokine produced by a variety of cell types, including macrophages and dendritic cells (DCs), as well as myeloid-derived suppressor cells.^11^^,^^14^ The heterodimeric IL-27 receptor is comprised of gp130 and IL-27Rα (WSX-1) and expressed on many cell types including T cells, natural killer cells, monocytes, DCs, B cells, and subsets of endothelial and epithelial cells.^15–18^ Upon the binding of IL-27 cytokine to the receptor on a naïve T cell, a JAK/STAT signaling cascade leads to polarization toward a Th1 phenotype.^19^^,^^20^ It was this role for which IL-27 was first identified; however, IL-27 also promotes STAT3 signaling, which can induce increased expression of the immune-suppressive cytokine, IL-10.^21–23^ Additionally, neonatal mice that lack IL-27Rα and cannot respond to IL-27, experienced improved control of bacteria with reduced inflammation and mortality compared with their wild-type counterparts.^24^^,^^25^ These data suggest IL-27 to be a significant factor in the early-life immune system that promotes an inadequate host response during infection. With the use of the IL-27 reporter mouse strain, an inconspicuous population of IL-27–producing DCs predictive of adjuvant-induced specific CD8^+^ T cell responses was identified.^26^ This finding highlights not only that it is possible, but also that it is essential, to identify and study IL-27 producers in an effort to expand what we know about this key population of immune-shaping cells.

These data suggest that IL-27 is an attractive therapeutic target in the wake of increasing antimicrobial resistance across the globe. It is essential to prioritize finding novel approaches to combat infections, especially for this highly vulnerable demographic. To advance IL-27 neutralization as a potential intervention strategy in the clinic for neonatal sepsis, we have to understand the characteristics of the cells that produce IL-27, the tissues that these cells are derived from, and how they may interact in the host response to infection. Previously, this was unknown and assumed from historical IL-27 biology.

In this body of work, we directly address the knowledge gap by specifically characterizing the cells that produce IL-27 in tissues with high levels of *E. coli* harvested from neonatal mice experiencing bacterial sepsis. Our laboratory’s well-established model of neonatal bacterial sepsis recapitulates the lethargy, poor feeding, dehydration, hypoglycemia, high bacterial burdens, and mortality observed in clinical cases of neonatal bacterial sepsis.^25^^,^^27^ We expanded our model to a transgenic mouse that expresses green fluorescent protein (GFP) under control of IL-27p28 regulation, resulting in a reliable reporter for IL-27–producing cells.^26^^,^^28^ Considering the previously described^25^ profound impact of IL-27 on disease outcomes during neonatal sepsis, the overall IL-27–producing populations from each tissue were surprisingly small. The liver displayed the highest number of IL-27 producers at baseline, and this population expanded in both the liver and lung during infection. We showed that IL-27 producers comprised a distinct population of cells that varied in expression of myeloid surface markers and unique subpopulations develop during infection. We observed important differences in quantity and between the cells comprising the IL-27 profile across tissues during neonatal bacterial sepsis. Single-cell sequencing of IL-27 producers in the liver demonstrated individual subpopulations and insight into the purpose these cells serve during infection. We observed that while all the cells were identified as IL-27 producers, their independent roles in contributing the neonatal immune landscape displayed a dynamic range of functions. This detailed profile is essential in understanding early-life immunity and IL-27 production in neonates.

## Materials and methods

### Animal care and use

Adult heterozygous IL-27p28eGFP mice were a generous donation from Dr. Ross Kedl at the University of Colorado Anschutz School of Medicine and maintained at West Virginia University (WVU) School of Medicine. These mice express enhanced GFP under control of the regulatory region of the IL-27p28 gene in the C57BL/6 background.^26^ Mating these mice resulted in both transgenic and nontransgenic littermates that were genotyped prior to experimental use. Mice were defined as neonates through 8 d of life.^11^^,^^13^ Both male and female pups were used for experimental infections. Pup sexes were visually determined similar to as described previously.^29^ All procedures were approved by the WVU Institutional Animal Care and Use Committee (protocol no. 1708008935) and conducted in accordance with the recommendations from the Guide for the Care and Use of Laboratory Animals by the National Research Council (2011).

### Murine sepsis infection model

*E. coli* O1: K1: H7 (ATCC) was engineered to express luciferase as described previously.^25^ A target inoculum of 5 × 10^5^ bacteria per mouse was prepared from an exponential growth culture of bacteria. Bacteria were washed with phosphate-buffered saline (PBS) and enumerated as described previously.^27^ Mouse pups were inoculated subcutaneously into the subscapular region with a volume of 50 µL of bacteria using a 28-gauge insulin needle on day 4 of life, as described previously.^25^ The mice were monitored twice daily for signs of morbidity and endpoint criteria established in prior literature.^25^^,^^27^ These include lack of mobility, inability to right themselves, >15% weight loss, and discoloration. Weights were collected prior to the infection and 18 to 20 h later before euthanasia. Blood was collected from control and infected pups in tubes containing 8 μL of 500 mM EDTA (Fisher Scientific). Solid tissues were collected in RPMI 1640 (Gibco) until processing.

### Whole-animal imaging

Pups were imaged with the IVIS SpectrumCT (PerkinElmer) available in the WVU Animal Model Imaging Facility. Signal intensity and location from the bioluminescent *E. coli* were recorded in both 2- and 3-dimensional formats. Signal was quantified by region-of-interest construction around the area of luminescence. Isoflurane was delivered through a custom nose piece developed by the WVU Animal Model Imaging Facility at 3% to 5% to accurately deliver the minimal level of anesthesia to prevent mice from repositioning during image acquisition. Images were processed and analyzed using Living Image (v. 4.7.4) (PerkinElmer). Luminescence was shown in radiance for 2-dimensional images and photons per second for 3-dimensional images.

### Tissue processing

Bacteria in the blood were enumerated by serial dilution and standard plate counts on tryptic soy agar (VWR). Approximately 1 to 2 µL of blood was used to measure glucose levels (mg/dL) with a glucometer and associated test strips (AlphaTrak3; Zoetis). Serum was obtained from the remaining blood by centrifuging the blood for 10 min at 2,000 *g* at 4 °C. Serum was collected and stored at −80°C until cytokine analysis.

Neonatal spleens were crushed in a 40 µm strainer (Greiner Bio-One) placed inside a cell culture suspension dish (Corning). Lungs and livers were transferred from the RPMI into tissue dissociation tubes (Miltenyi Biotec) containing dissociation enzymes. Neonatal lungs were dissociated using the Miltenyi Biotec GentleMACS Octo Dissociator with Heaters, located in the WVU Flow Cytometry and Single Cell Core Facility (WVU FCSCCF), using the 37C_m_SDK_1 protocol and the Miltenyi Biotec murine lung dissociation kit. Neonatal livers were dissociated using the same instrument with the 37C_m_LIDK_1 protocol and the Miltenyi Biotec murine liver dissociation kit. Following dissociation, tissues were pelleted at 350 *g* for 5 min at 4 °C and ACK lysed for 4 min at room temperature. ACK was neutralized with 10% fetal bovine serum in RPMI and pelleted at 350 *g* for 5 min. Cell debris was removed by putting cells through 40 µm strainer in a 50 mL conical tube and pelleted. Cells were counted and assessed for viability using the Thermo Fisher Scientific Countess 3 Automated Cell Counter.

### Serum cytokine detection

Serum collected from PBS control or *E. coli*–infected pups as described previously was diluted 1:2 per manufacturer guidelines. Serum was plated on a U-plex multiplex electrochemiluminescence assay (Meso Scale Discovery) detects the IL-27p28 subunit. Reagents were prepared following the manufacturer protocol and incubations were performed as prescribed. The plate was read with the MSD QuickPlex SQ120 available in the WVU FCSCCF. Data were analyzed with Discovery Workbench (v. 4.0).

### Flow cytometry

The cells isolated from control or infected mice as described previously were quantified and 2 × 10^6^ cells were labeled with the myeloid panel shown in Table 1. Cells were incubated with FcR blocking reagent (Miltenyi Biotec) and labeled with the antibody panel covered on ice for 1 h and washed twice before fixation with 0.4% paraformaldehyde. Cells were analyzed with a BD LSRFortessa using the BD FACSDiva v. 9.2 software. A minimum of 3 × 10^5^ events were collected for the spleen, liver, and lung. Analysis was performed using FCS Express 7 (De Novo Software). The cell population (forward scatter area vs. side scatter area) for each tissue was gated for singlets (forward scatter area vs. forward scatter width) and then for CD45 vs. viability stain (FVS780, 1:3,000; BD 565388) to define live immune cells for each tissue (Figs. S1–S3). To identify the IL-27–producing cells, CD3/CD19 vs. GFP was used to exclude the lymphoid cells and visualize the myeloid GFP^+^ population (Figs. S1–S3). Uniform Manifold Approximation and Projection (UMAP) analysis of the markers within the live immune population was performed following resources available through De Novo software.^30^^,^^31^

**Table 1.** Myeloid cell antibody panel.

Marker	Quantity	Stain/Conjugate	Clone	Catalog
Ly6G/C	0.125 µg	APC	RB6-8C5	BD 553129
F4/80	0.125 µg	PE	T45-2342	BD 565410
CD45	0.125 µg	R718	30-F11	BD 567075
CD11b	0.125 µg	BV786	M1/70	BD 740861
CD11c	0.25 µg	BV510	N418	BD 744178
MHCII	0.25 µg	BV650	Tu169	BD 563415
CD3	0.25 µg	PE-CF594	145-2C11	BD 562286
CD19	0.25 µg	PE-CF594	1D3	BD 562291
CD204	0.25 µg	BB700	268318	BD 748090

MCH, major histocompatibility complex class II.

### Single-cell RNA sequencing and Gene Ontology analysis

The livers of control or infected neonatal mice were pooled and homogenized as described above. The cells were gated for the live CD45^+^/CD3^−^/CD19^−^/GFP^+^ population, as shown in Fig. S2, and sorted using the BD FACSAria III available in the WVU FCSCCF. Downstream steps were performed according to the 10x Genomics manufacturer recommendations for the 10x Genomics Chromium Next GEM Single Cell 3′ Kit v3.1 (Product 1000269). The library was prepared using the 10x Genomics Chromium Controller available in the WVU FCSCCF by the WVU Genomics Core Facility. Sequencing was performed using the Illumina NextSeq 2000 sequencer available at Marshall University. Data quality control, normalization, scaling, variable gene identification, and clustering were performed using the Seurat R package (v. 5) and custom R code. Cell data quality control and filtering was based on cellular complexity (number of genes per cell) and mitochondrial reads. Viable cells were selected by filtering for cells with a UMI count between 500 and 2,500. Cells with >200 genes and mitochondrial percent reads <40 were kept for analysis. Following quality control analysis and data filtering, there were 622 control cells and 1,016 infected cells.

After data normalization and scaling, Principal component analysis (RunPCA) were performed on the 2,000 most variable genes (FindVariableFeatures). Quality control metrics are visualized in Fig. S4. The RunHarmony function from the harmony R package was used to correct for potential batch effect, and Harmony embeddings were used to define the dimensionality of our dataset. The resulting number of principal components were used in the RunUMAP function for reduction into 2 dimensions and for nearest neighbor graph (SNN) creation (FindNeighbors). The Clustree R package was used to compute the optimal resolution for clustering (res = 1.1) (Fig. S5), which was then supplied to the FindClusters function. Differentially expressed genes (DEGs) between infected and control cells were calculated with FindMarkers and cluster-specific gene signatures were obtained by FindAllMarkers. Cells were annotated using the Cell Typist^32^ with the Healthy_Mouse_Liver model and Majority Voting mode ON.^33^

DEG lists (*Mus musculus*) of the top 4 cell type clusters (Kupffer cells [KCs], monocytes and derived cells, neutrophils, and migratory conventional DCs [cDCs]) were loaded into Metascape^34^ for Gene Ontology analysis. The top 20 pathways for increased or decreased genes from each cluster were shown. Analysis was performed using the Express analysis settings available through Metascape.

### Data accessibility

All raw sequenced data are publicly available at the Gene Expression Omnibus under accession number GSE283847. Secure token for data access during manuscript peer review is gtmbqsiytjujbir (https://www.ncbi.nlm.nih.gov/geo/query/acc.cgi?acc=GSE283847).

### Statistics

Statistical significance was determined with the appropriate parametric or nonparametric test for each of the datasets as described in the figure legends. Data were analyzed as either mean or log_10_-transformed data ±SEM using correlations, unpaired *t* tests, Mann-Whitney tests, and/or 2-way analyses of variance. Statistical significance was analyzed using GraphPad Prism 10 (GraphPad Software). The threshold for significance was set to alpha = 0.05.

## Results

### Septic neonatal mouse physiological measurements are consistent with human disease and correlate with the magnitude of bacterial burden

**Figure 1.**
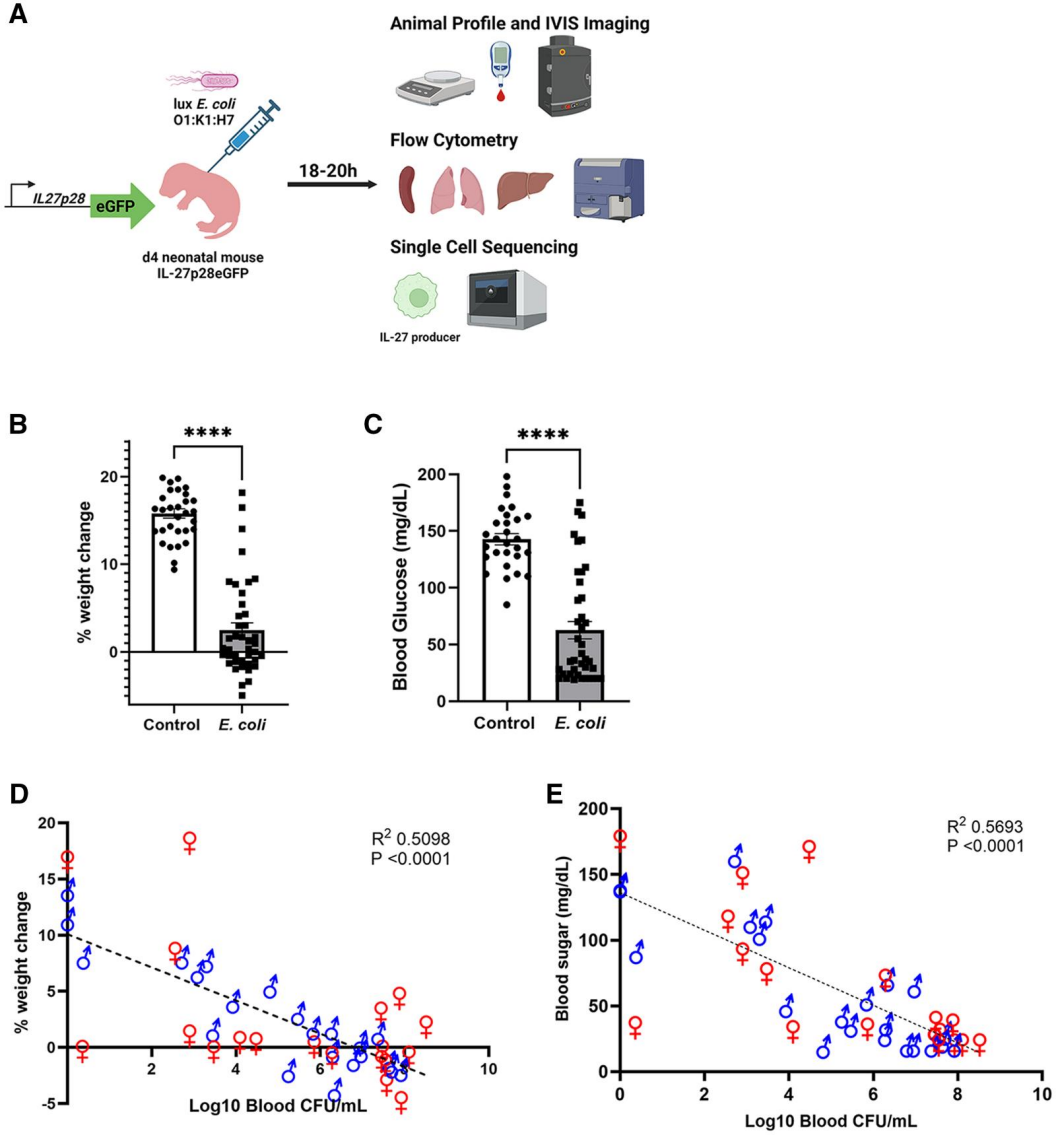
Timeline schematic of infection and animal infection profile. (A) The study design, workflow, and analysis are illustrated. IL-27p28eGFP mice were subcutaneously inoculated with a target of 5 × 10^5^ CFUs/mouse of luciferase-expressing hemorrhagic *E. coli* for 18 to 20 h. (A) Mice were weighed, blood glucose was recorded, and mice were imaged using IVIS-CT. Spleens, lungs, and livers were taken for flow cytometry, and IL-27–producing cells sorted from the liver were analyzed by single-cell sequencing. Created with BioRender.com. (B) Mean percent change in weight ± SEM (****P < 0.0001) and (C) mean blood glucose ± SEM (****P = 0.0012) from 0 h to 18 to 20 h was recorded from PBS control (n = 32) vs *E. coli*–infected (n = 41) animals. (B, C) Each animal is represented with an individual symbol. Statistical significance was determined using a Mann-Whitney test. (D) Log_10_ blood CFUs/mL were measured in the infected mice at 18 to 20 h and correlated with percent weight change or (E) blood glucose value collected at the same time. (D, E) Each data point represents an individual mouse (n = 41). Significant correlations were determined using a Pearson correlation test.

It is known that neonatal mice express elevated levels of IL-27, but a complete description of the cells responsible for its production in the presence and absence of infection is lacking. Previously, we and others have described heterogeneous populations of myeloid cells as IL-27 producers^11^^,^^20^^,^^21^^,^^35^; however, a detailed in vivo analysis of paired single-cell phenotypic and transcriptional data specifically in neonatal mice during sepsis has not been reported. In the studies presented here, we followed our standard infection strategy to investigate IL-27 producers during neonatal bacterial sepsis by IVIS-CT, flow cytometry, and single-cell sequencing (Fig. 1A). We found that pups infected with luciferase-expressing *E. coli* O1:K1:H7 failed to gain weight as effectively as control animals and became hypoglycemic (Fig. 1B, C). There was a mean 2.56 × 10^7^ ± 8.35 × 10^6^ (SEM) colony-forming units (CFUs) per mL of *E. coli* present in the blood from infected pups. The lack of weight gain and decreased blood sugar values correlated significantly with bacterial burden in the blood (Fig. 1D, E). These data are consistent with previously reported findings using this model and further demonstrate the translational relevance.^25^^,^^27^^,^^36^^,^^37^

### The liver is an increased source of IL-27 during infection

**Figure 2.**
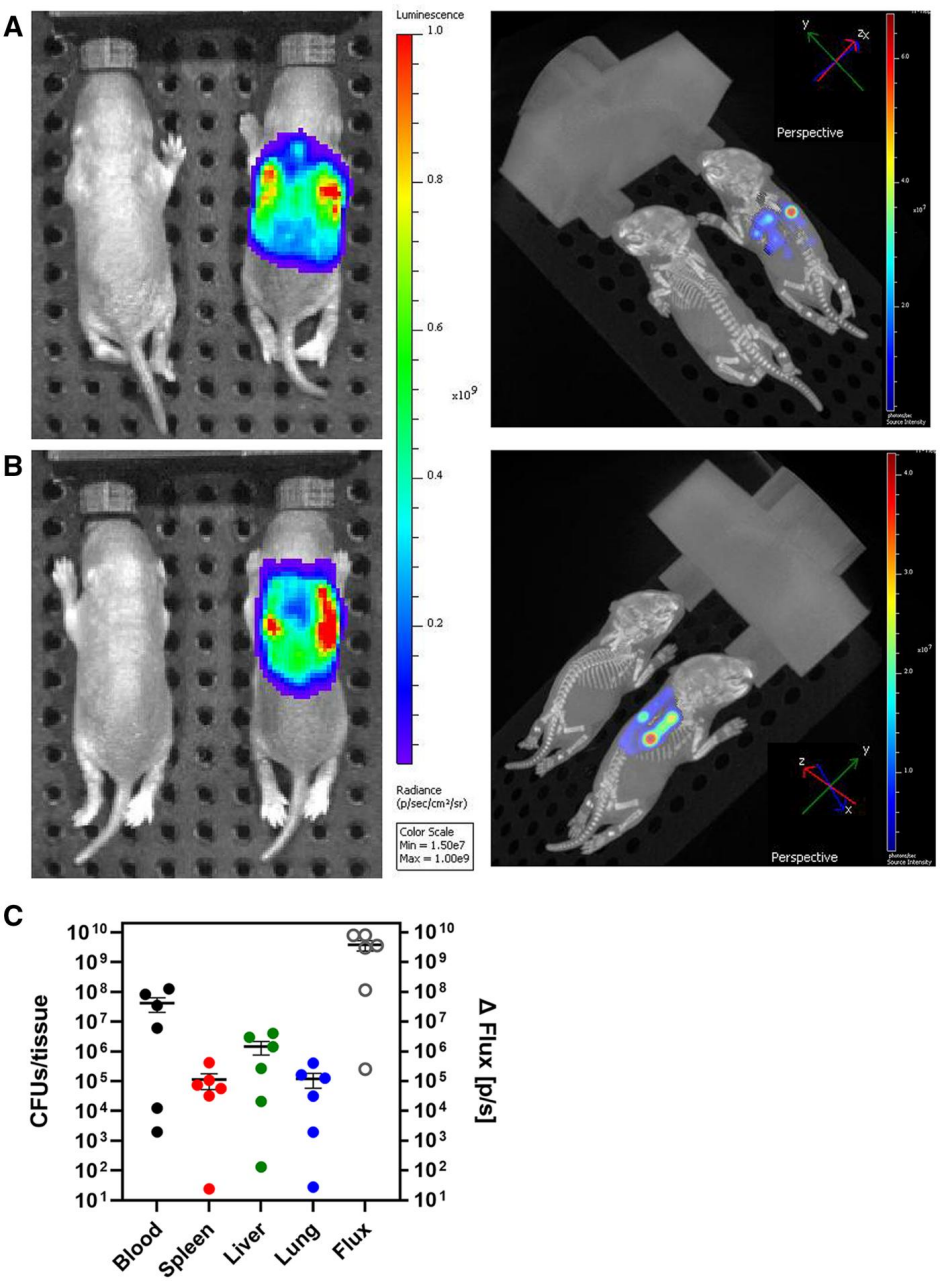
Bacterial luminescence measured by IVIS-CT demonstrated high tissue burden in the spleen, liver, and lung. (A, B) Representative images of 2 sets of control (left) and *E. coli*–infected (right) pups at 18 h postinfection in 2-dimensional with 3-dimensional orientations to show the (A) left and (B) right sides of the mice. (C) The mean bacterial burdens ± SEM in the blood (CFUs/mL, black), spleen (red), lung (blue), and liver (CFUs/tissue, green) in addition to the change in flux (photons/second, gray open circle) for mice assessed by IVIS-CT at 18 h postinfection were reported (n = 6). Each symbol within a tissue represents an individual mouse.

In prior work, we have identified the lung, liver, kidney, brain, and spleen to be sites of infection in which there are measurable bacterial burdens.^25^ We hypothesized that IL-27 production would be driven at least in part by bacterial burden, and as such, performed whole-animal imaging of pups to identify anatomical regions that displayed high levels of bacterial luminescence. This allowed us to have a directed approach when choosing tissues to analyze for IL-27–producing cells. Representative images with some expected spatial heterogeneity in individual mice display bilateral luminescence consistent with localization to the lungs and additional signal in proximity to the spleen and liver (Fig. 2A, B). Following necropsy, standard plate counts showed high bacterial burdens in the blood, spleen, liver, and lung in support of the luminescence interpretation (Fig. 2C). Blood burdens were represented as CFUs/mL and solid tissues were split evenly for burdens (CFUs/tissue) or flow cytometry. Flux is a measurement of luminescent brightness, measured in photons per second, which was also paired with the bacterial burdens from the tissues (Fig. 2C).

**Figure 3.**
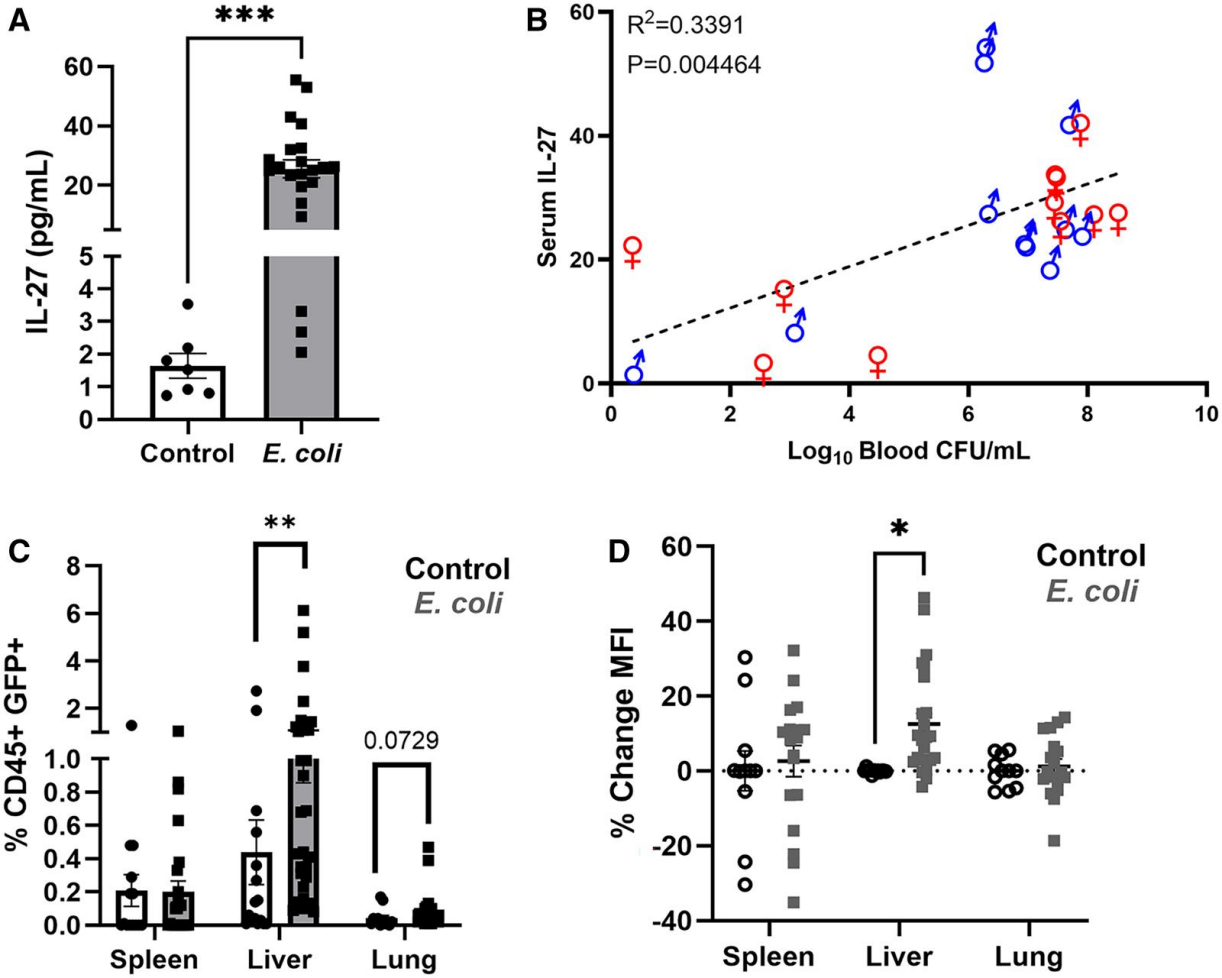
Serum IL-27 increases with infection and high-bacterial-burden tissues contain IL-27 producers. (A) IL-27 levels were measured by MSD electrochemiluminescent immunoassay from the serum of control (n = 7) or *E. coli*–infected (n = 22) pups collected at 18 to 20 h postinfection; data analyzed using an unpaired t test (***P < 0.0001). (B) IL-27 levels correlated with the log_10_-transformed blood CFUs/mL in the infected animals at 18 to 20 h postinfection using the Pearson test (R^2^ = 0.3676). (C) The mean percent of live CD45^+^ myeloid GFP^+^ cells (#GFP^+^/#CD45^+^ × 100) ± SEM gated as described in the Materials and Methods for each tissue were analyzed using a Mann-Whitney test and graphically represented together (**P = 0.0021; lung *P* = 0.0729). (D) The mean percent change of median fluorescence intensity (MFI) of live CD45^+^ myeloid GFP^+^ IL-27 producers. The MFI for each control and infected replicate relative to the experimental control sample mean was recorded for each individual neonate within a tissue. Statistical significance between control and infected within a tissue was determined using an unpaired *t* test with a Welch’s correction (*P = 0.000234).

In this representative experiment and subsequent others, the lung, liver, and spleen were additionally analyzed for GFP expression to compare the IL-27 profile between the control and infected pups. Consistent with our previous reports, there was a significant increase in IL-27 serum levels that was observed during infection in comparison to control (Fig. 3A). There was a positive relationship observed between the level of IL-27 in the serum and the bacterial burden observed in the blood (Fig. 3B). In order to visualize the GFP^+^ population that represents IL-27 producers, we targeted the live nonlymphoid immune population (CD45^+^/CD3^−^/CD19^−^/GFP^+^) (Figs. S1–S3). Analysis of the lymphoid (CD45^+^/CD3^+/−^/CD19^+^) cells did not reveal a meaningful number of GFP^+^ cells (Figs. S1–S3). Surprisingly, given the dramatic increase in circulating IL-27 at 20 h following infection, the abundance of IL-27 producers amongst the total CD45^+^ population in all tissues examined was smaller than expected. While there were no significant differences between control and infected spleens or lungs, the livers displayed significant increases in GFP^+^ cells, indicative of more IL-27 production (Fig. 3C). To determine whether or not the GFP^+^ IL-27 producers in some tissues may also express more protein per cell during infection, we examined the percent change in the median fluorescence intensity of the GFP^+^ population in the infected animals relative to the signal recorded in the PBS control animals within each experiment. Indeed, IL-27 producers in the liver were more consistently responsible for a greater level of fluorescence (Fig. 3D). Together, we were able to employ a targeted tissue approach to explore IL-27 expression based on bacterial signature visualized by whole animal imaging (Fig. 2) and identified a changing dynamic of IL-27 production in the liver during infection that warranted further analysis of those cell populations (Fig. 3C, D).

### IL-27 producers vary across tissues

**Figure 4.**
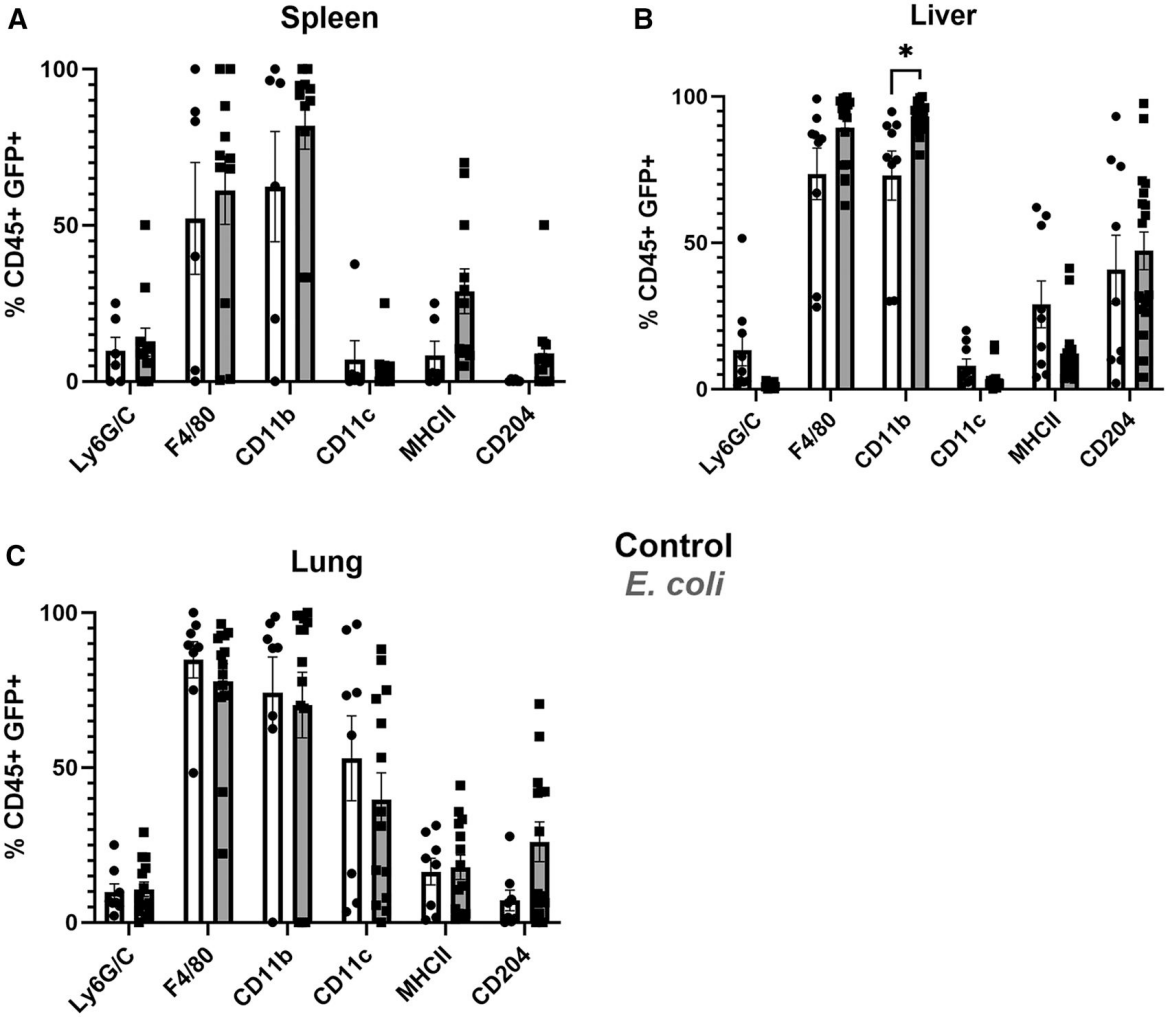
IL-27 producers within tissues vary in the expression of myeloid markers. The percent of cells within the live CD45^+^ myeloid GFP^+^ population of control (white) or *E. coli*–infected (gray) animals were positive for surface expression of myeloid markers (Ly6G/C, F4/80, CD11b, CD11c, major histocompatibility complex class II, and CD204) gated as described in the Materials and Methods and shown for (A) spleens, (B) livers, and (C) lungs. Mean ± SEM of the individual marker populations were analyzed for statistical significance using 2-way analysis of variances and Šidák’s multiple comparisons test (*P = 0.0301).

Following the identification of IL-27 producers in peripheral tissues, we wanted to further understand the GFP^+^ cell phenotypes. We chose a common set of markers used in prior reports to identify myeloid populations.^38^^,^^39^ The understanding of cells specifically responsible for IL-27 during neonatal bacterial sepsis is incomplete. When observing the phenotypes represented within the live CD45^+^ myeloid (CD3^−^CD19^−^) GFP^+^ population within the spleen, liver, and lung, GFP^+^ leukocytes were most commonly F4/80^+^CD11b^+^ with significant differences in the frequency of CD11b expression between control and infected animals in the liver (Fig. 4A–C). F4/80 expression on GFP^+^ cells was most abundant in the lung and liver (Fig. 4B, C). Generally, there was a modest frequency of Ly6G/C^+^ cells (Fig. 4A–C). The frequency of major histocompatibility complex class II expression on IL-27 producers was low in all tissues; however, there were some interesting trends when comparing the spleen and liver. While statistical significance was not achieved, there was an increased frequency of major histocompatibility complex class II expression on IL-27 producers in the spleen during infection while the inverse relationship was found in the liver (Fig. 4A, B). The myeloid marker expression did not change between control and infected in the lung (Fig. 4C). IL-27 producers from the lung expectedly displayed the greatest CD11c signature, a marker commonly associated with alveolar macrophages,^40^ relative to the other 2 tissues. Interestingly, the liver showed the highest frequency of CD204^+^ cells at nearly half the IL-27–producing population (Fig. 4B).^41^^,^^42^

**Figure 5.**
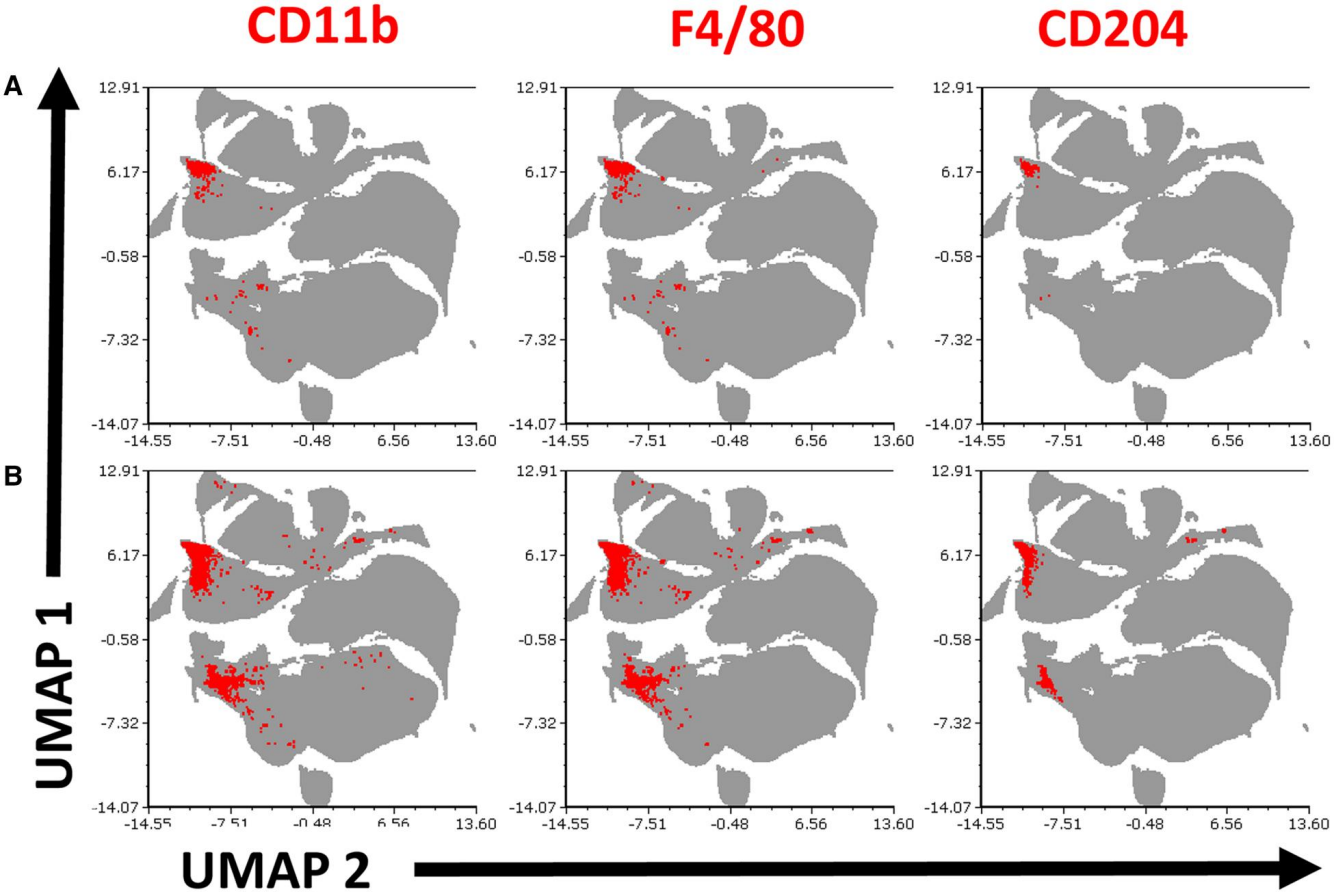
IL-27 producers from the liver exhibit different surface phenotypes for control vs. infected animals. (A) The total live CD45^+^ population (gray) of the liver was shown with GFP^+^ cells expressing CD11b, F4/80, or CD204 (red) from a representative experiment were analyzed by unsupervised UMAP. Cells from (A) control and (B) *E. coli*–infected mice were displayed.

The expression profile of individual markers was informative about IL-27 producer phenotypes; however, a cell is not limited by expression of a single marker. As such, we hypothesized that subpopulations with various coexpression of surface markers could emerge during infection. To address this, we analyzed combinations of markers on individual GFP^+^ cells via unsupervised UMAP in FCS Express. Across several experiments, we observed an expansion of clusters present at baseline following infection as well as the emergence of unique cellular subpopulations within the liver (Fig. 5). This suggests that depending on the tissue, location within the tissue milieu, and additional signals during infection, heterogeneous subpopulations of IL-27 producers expand.

### Myeloid subpopulations display differential gene expression during infection

**Figure 6.**
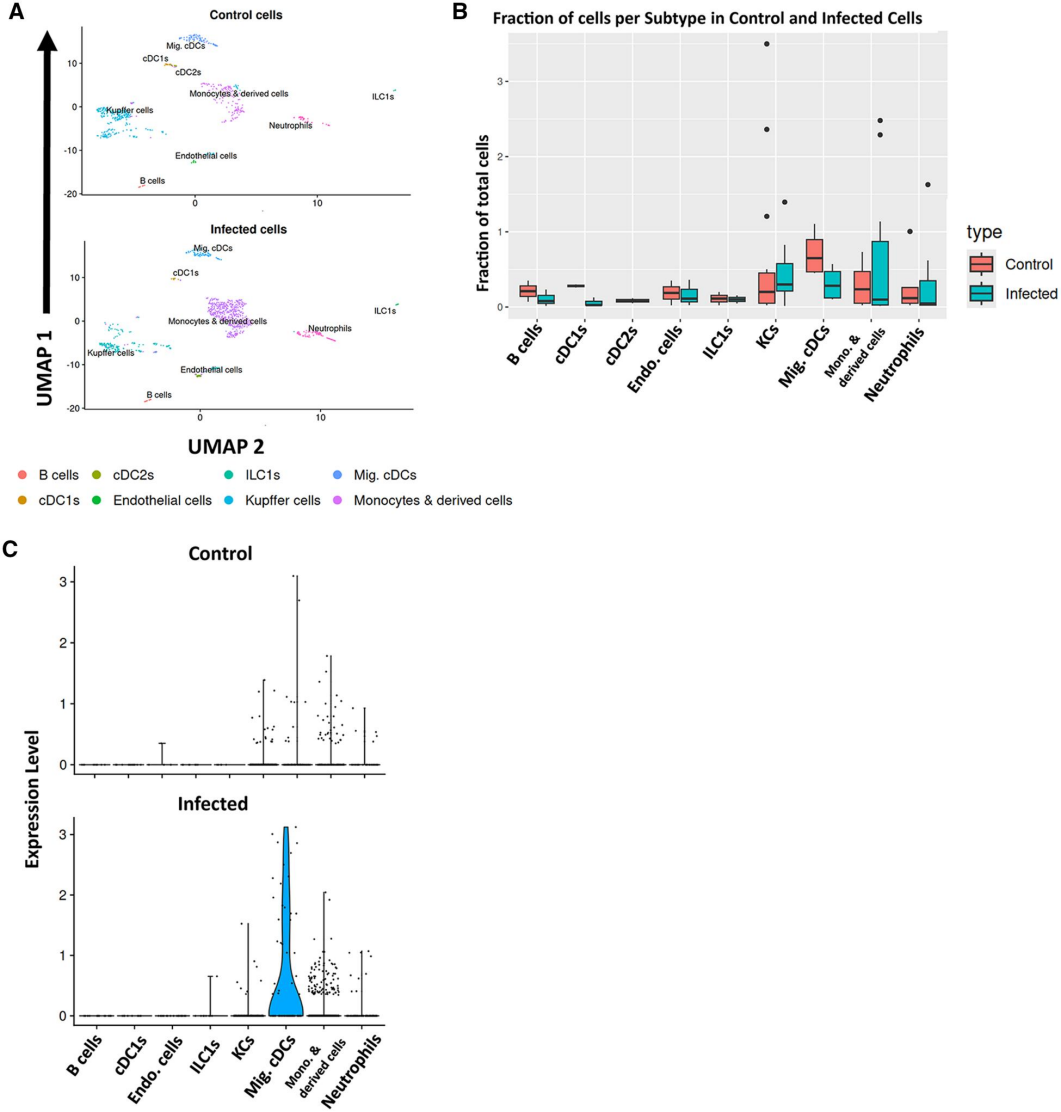
Single-cell profiles of control and infected IL-27 producers in the liver. (A) Single-cell profiling revealed cell clusters in the liver of control (top) and *E. coli*–infected (bottom) neonates at 18 h postinfection annotated and visualized by UMAP. (B) The fraction of total sorted and sequenced liver cells per cell type cluster were compared for control (orange) and *E. coli*–infected (blue) neonates. (C) The IL-27p28 expression level based on RNA sequencing between control (top) and *E. coli*–infected (bottom) cells for each cell type cluster was visualized by a violin plot.

To further validate the phenotypic analysis of IL-27 producers and begin to understand more about their functionality, we performed 3′ single-cell RNA sequencing. With the expectation that the number of GFP^+^ cells would be a limiting factor in other tissues, we sorted cells from the livers from 2 control and 2 infected animals to obtain the live CD3^−^/CD19^−^/CD45^+^/GFP^+^ (Fig. S2) population for barcode labeling by the 10x Chromium Controller. Following sequencing, we observed several cell types, which predominantly included KCs, monocytes and monocyte-derived cells, subpopulations of cDCs, and neutrophils based on gene expression (Fig. 6A). Despite the exclusion of CD19 expressors, we identified a very minor population annotated as B cells that escaped gating. There were modest differences in the fraction of cells within each subtype between control and infected; higher variability was observed among infected cells than control (Fig. 6B). Notably, the samples from infected neonates had an expansion of KCs with a shift in the UMAP accompanied by additional increases in monocytic cells and neutrophils (Fig. 6A, B). The infected migratory cDC population was decreased relative to the control population but was responsible for the most significant levels of active IL-27p28 expression, and overall, the infected cells expressed more IL-27 than the control (Fig. 6B, C). These data further supported the phenotyping results.

**Figure 7.**
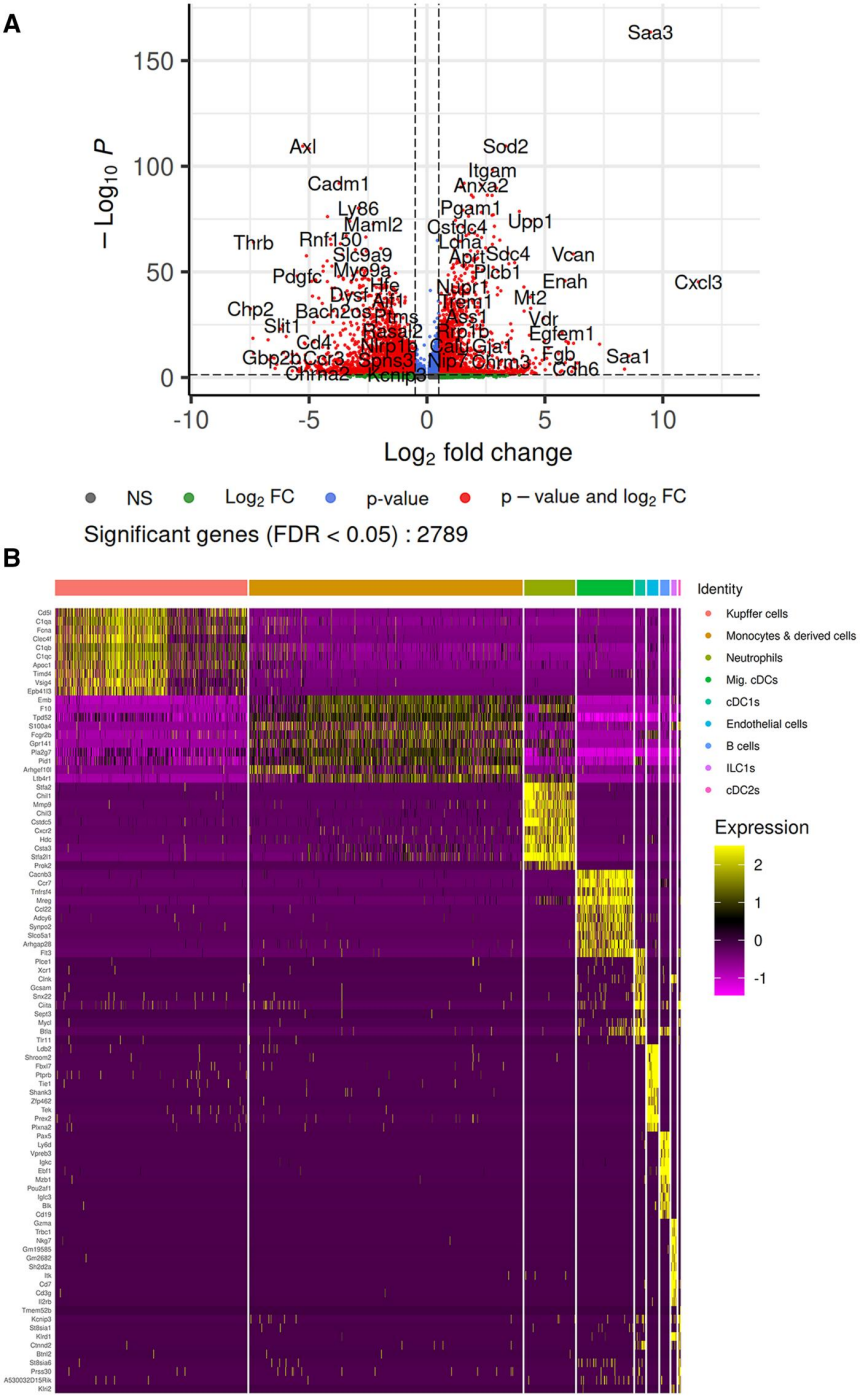
IL-27–producing cells within different subclusters express different genes. (A) Log_2_ fold change (FC) of the DEGs in the *E. coli*–infected cells isolated from the liver at 18 h postinfection relative to the control were visualized by volcano plot (false discovery rate < 0.05, 2,789 genes). Shown in red are the genes with a statistically significant log_2_ FC (B) The expression levels of the top 10 DEGs for individual cells within each cell type in the infected cells relative to control was shown via heatmap. Each cell within the colored bar annotated cluster is represented by a separate column and each gene by a separate row. The expression values for the gene of each cell are *z*-score transformed according to color as shown in the legend.

An analysis of DEGs during infection identified 1,034 genes that were increased and 1,755 genes that were decreased (Fig. 7A). Gene expression patterns suggested high levels of inflammation during sepsis in the infected cells relative to the control (Fig. 7A). Specifically, genes encoding serum amyloid A, an acute phase protein and sepsis biomarker, were among the highest expressed genes during infection.^43^^,^^44^ The gene for the neutrophil-recruiting chemokine CXCL3 was also increased with infection, whereas CCR3, associated with monocyte recruitment, was decreased (Fig. 7A). PGAM1 (phosphoglycerate mutase 1), which also fell among the increased genes, catalyzes the reversible reaction of 3-phosphoglycerate to 2-phosphoglycerate in glycolysis and gluconeogenesis. (Fig. 7A).

The top 10 highest expressed genes for each cell type cluster were used to compare differences in gene expression levels across clusters (Fig. 7B). The leading genes within the subclusters varied, suggesting functional differences between the cells within each cluster. The KCs expressed scavenger receptor (*CD5l*, *Fcna*, *Clec4f*, and *Timd4*) and complement-related genes (*C1qa*, *C1qb*, *C1qc*, and *Vsig4*) at a high level (Fig. 7B). Monocytes and derived cells expressed a collection of genes with roles in inflammatory responses and cell signaling (*S100a4*, *Ltb4r1*, *Pla2g7*, and *Pid1*). Together, these data identified specific populations of cells responsible for making IL-27 during infection and how those populations change along with transcriptomic differences that occur during infection.

### Gene Ontology enrichment analysis yielded dynamic functions of DEGs from cell clusters

**Figure 8.**
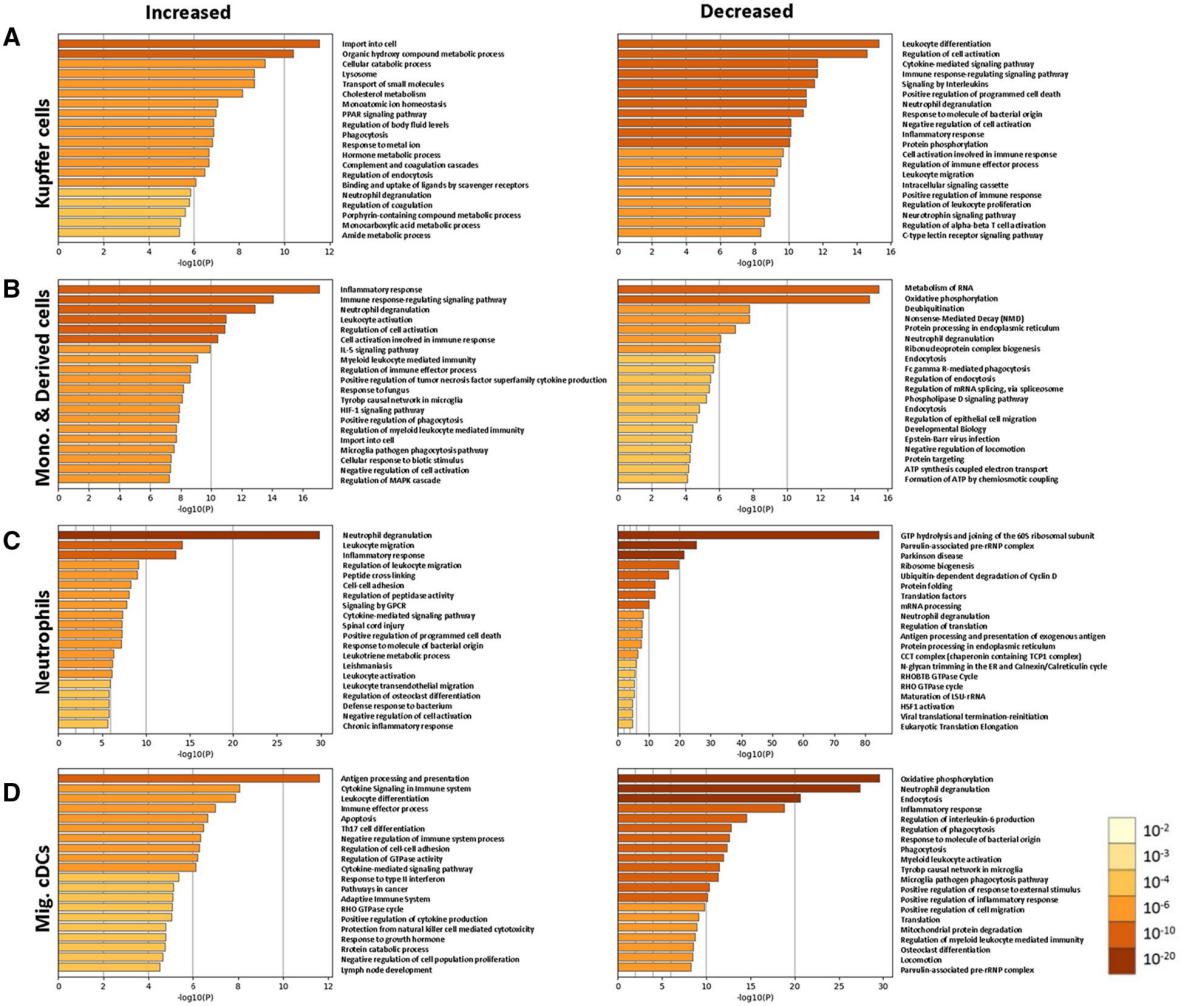
Subclusters of IL-27 producers perform different functions. DEGs in the liver at 18 h postinfection for (A) KCs, (B) monocytes and derived cells, (C) neutrophils, and (D) migratory cDCs were subjected to pathway analysis through Metascape. The top 20 pathways and corresponding degree of enrichment for increased (left) or decreased (right) DEGs during infection within each subcluster are shown.

GO enrichment analysis was performed to assess the hypothesis that not all IL-27–producing cells are functionally equivalent. The top 20 pathways for differentially increased or decreased genes in the top 4 clusters are shown in Figure 8. Together, the diversity within these cells may have the potential to dictate the outcome of infection by positively and negatively affecting immune responses. The data suggest a KC response to bacteria evidenced by pathways related to cellular imports, phagocytosis, lysosomal activity, and scavenger receptor activity (Fig. 8A, left). Metabolism of cholesterol may represent a cellular response to a limited glucose environment (Fig. 8A, left). Decreased pathways included a variety of means of cellular communication and regulation, including cytokine and intracellular signaling, differentiation of other cells, responses to other cells, and a reduced inflammatory response (Fig. 8A, right). Monocytes and derived cells reflect enhanced responses to inflammatory and stress signals with increased activation and intracellular signaling within the population (Fig. 8B, left). Upregulation of HIF-1–associated genes concomitant with reduced expression of genes involved in oxidative phosphorylation may reflect tissue necrosis and reduced oxygen availability (Fig. 8). In contrast to KCs, the monocytes and derived cells reduced expression of phagocytic, endocytic, and post-translational modification-related processes (Fig. 8B, right). Neutrophils appeared to be newly recruited with a commitment to increased expression of cell adhesion and migratory genes, as well as highly inflammatory based on the notable enrichment of genes affiliated with neutrophil degranulation and inflammatory responses (Fig. 8C, left). Protein synthesis and processing was downregulated in neutrophils, decreasing alongside homeostasis-related functions that may be the result of endoplasmic reticulum stress (Fig. 8C, right). The migratory cDC population, which had the most IL-27 expression in Figure 6, also appeared the least inflammatory while downregulating antigen uptake pathways and increasing presentation gene sets (Fig. 8D).

## Discussion

In this study, we sought to identify the specific tissue and cellular sources of IL-27 alongside the transcriptional signature for these cells. We believe that IL-27 antagonization represents a potential therapeutic approach to expand the physician toolbox when treating cases of neonatal bacterial sepsis. While this is more likely to represent an approach that augments antibiotics than a stand-alone therapy, the value could still be immense. IL-27 antagonization might reduce the effective concentration of antibiotics clinically, and it may increase their effectiveness with bacteria that demonstrate intermediate and/or broad antimicrobial resistance. To develop this approach effectively, it is first critical to understand where this immunosuppressive cytokine is already elevated and continues to increase during infection. We utilized IL-27p28eGFP mice in our previously established murine model of neonatal bacterial sepsis to determine tissues in which IL-27 producers are located during infection, the specific cell types responsible for IL-27 production, and the gene expression profile and functions that those cell populations have within our model. Additionally, a deeper understanding of the tissues from which IL-27 producers are derived is important for pharmacological considerations and effective therapeutic design.

In the IL-27p28eGFP reporter mouse, IL-27–producing cells express GFP and are tagged for identification and sorting. We observed the same clinically relevant infection outcomes that we have previously reported for C57BL/6 mice.^25^^,^^27^ We chose the 18- to 20-h window for our investigation of the IL-27 producer profile, as we have previously shown that both IL-27 subunits are well expressed in peripheral tissues in the first 24 h postinfection, serum levels peak in this model at 24 h postinfection, and this time period is a critical window for control of the bacterial burden within the tissues and corresponding survival during infection.^25^ While GFP is reflective of IL-27 expression, the fluorescent protein also requires an accumulation threshold for accurate detection. We showed significant levels of hypoglycemia during infection, which is a routine clinical observation and described with our model,^37^^,^^45^^,^^46^ and high levels of IL-27 in the sera of mice infected with *E. coli* in line with our prior report.^11^ We observed a range of IL-27 in the serum during infection; however, we also found that serum values correlated positively with the bacterial burden in the blood. There was some variability in severity of infection within each animal depending on individual factors such as initial weight at time of infection, inoculum, and duration of infection, but collectively our data suggest that circulating levels of IL-27 are linked with the burden of bacteria in the blood.

We wanted to know where and when IL-27 levels increase to detail the identity of the IL-27 producers during neonatal bacterial sepsis, so we performed IVIS-CT and identified the spleen, liver, and lungs, all tissues that have been shown previously in our model to harbor bacterial burdens and express IL-27 transcripts.^25^ The spleen is a secondary lymphoid organ and critical for removal of encapsulated bacteria, so we hypothesized that its role as a venue for frequent interactions between *E. coli* and IL-27 producers. The liver is a metabolic hub responsible for filtering the blood and serves as a reservoir for immune cells, such as KCs, DCs, and natural killer cells.^47^ Metabolic activity is key in development of the neonatal body, which lacks fat reserves, while undergoing a significant change in nutritional content and mechanism of delivery, on top of a high energy requirement.^48^ The murine neonatal liver is also an important immune tissue, as primary hematopoietic functions transition from the liver to the bone marrow shortly after delivery and display a rich myeloid population.^49^^,^^50^ Neonatal KCs have further been shown to be in suboptimal position in the sinusoids for clearing *E. coli* infection.^51^ Additionally, neonatal bacterial sepsis patients often experience respiratory distress that can escalate to pneumonia or respiratory distress syndrome. This knowledge, combined with the upper thoracic region consistently displaying luminescent signal by IVIS-CT, meant that we chose the lung as a candidate for IL-27 producer profiling. We were initially surprised by the ≤3% average frequency of GFP^+^ cells indicating IL-27 production in any tissue; however, this may be a testament to the regulatory potential of a small population of cells.^26^^,^^28^ We found increased frequency of GFP cells in both the liver and lung; however, there was only an increase in the percent change in the median fluorescence intensity of that population in the liver. From the GFP^+^/IL-27 producer population, we used individual markers to phenotype the GFP^+^ cells and found that all 3 tissues possessed a high percentage of CD11b^+^ and F4/80^+^ cells.

Broadly, CD11b^+^ cells in the spleen are indicative of macrophage, monocyte, granulocyte, and DC populations. In the liver, the resident macrophage population, KCs, lack Ly6G but express CD11b and F4/80.^52^ The complete repertoire of phenotypic markers considered in the UMAP further suggests the appearance of a novel subpopulation of CD204^+^ KCs (Fig. 5B). Although the percent of CD204^+^ among total GFP^+^ cells was not statistically different between naïve and infected (Fig. 4B), the total number of GFP^+^ cells increased in the liver (Fig. 3C), translating to a gain of CD204^+^ cells observed during infection (Fig. 5). Previous reports have shown the emergence of a CD204^+^ population that appears to be beneficial during septic shock and in resolving excessive inflammation.^41^ This precedent, albeit from an adult model, suggests the CD204^+^ population to be suppressive. Importantly, elevated CD204 expression has been associated with worse outcomes in several types of cancer.^53–56^ Differential expression of scavenger receptor genes was evident in KCs (Fig. 7B) and enriched by GO analysis (Fig. 8A, left).

In addition to F4/80 and CD11b, the lung showed a higher frequency of CD11c^+^ cells than the spleen or the liver. While alveolar macrophages are generally considered CD11b^−^/CD11c^+^, interstitial macrophages express CD11b but not CD11c, so we expect these markers to reflect a heterogeneous combination of alveolar macrophages, monocytes and dendritic cells.^57^^,^^58^ Despite the lack of statistical significance of individual markers, we were able to see population shifts during infection and the emergence of subpopulations, as referenced previously, by UMAP. This speaks further to the importance of an in-depth analysis of the cells that produce IL-27 in early life and contradicts the notion of a static singular cell type that contributes the cytokine.

We focused on the liver for single-cell analysis because the highest level of IL-27 producers were found in this tissue, we saw the emergence of unique populations based on combinations of phenotypic markers (Figs. 3–5), the liver is important for maintaining metabolic homeostasis, and the liver has a unique role in early-life immunity. While it is a limitation of this study that we were not able to profile other tissues at the single-cell level for a systemic infection, the GFP population in the spleen and lung did not support feasibility of this approach. From these data, we learned that there are key differences in the IL-27–producing populations of control and infected cells in the liver. Figure 5 shows 2 populations emerging with infection, and this was reinforced by the transcriptome (Fig. 6A). We saw that the KCs in the infected mice were more homogeneous than control mice, with a regional shift in clustering. Combined phenotypic and transcriptional analyses further highlighted increased scavenger receptor expression as a key theme assigned to KCs during neonatal sepsis. We saw CD204 on the surface of the cells, scavenger receptor–related genes among the top genes for KCs, and their enrichment in GO analysis of all DEGs from KCs.

Across all cell types, we observed gene expression profiles reflective of increased inflammation. Others demonstrated that expression patterns linked wound healing, infection resolution, communication, and cell death. As the infection had not yet been resolved, this appeared characteristic of the dysregulation of the immune system observed during sepsis. We saw that the cells within the clusters expressed a range of genes with varying, and in some cases, oppositional functions, further suggestive of dysregulation, and also that IL-27 production comes from a diverse group of cells. Additionally, IL-27 producers from infected animals exhibited differences from that of control animals in surface markers, cell types, and gene expression. We observed different functional attributes between cell populations with Gene Ontology enrichment analysis. Together, the different functions among these cells could at first glance be considered an attempt to control the immune response to ameliorate the infection; however, this is a delicate balancing act, especially for the inexperienced neonatal immune system. Overall, a profile of IL-27 producers was suggestive of failed coordination and dysregulation of the immune system that typifies sepsis.

The data presented in this body of work suggested that KCs in neonatal sepsis are leading IL-27 producers based on their abundance amongst the GFP^+^ population. When considering the flow cytometry data with the Gene Ontology analysis, scavenger receptors become a common theme, as mentioned previously. While the gene for CD204 (*msr1*) is not among those increased within the binding and uptake of ligands by scavenger receptors pathway, it was interesting to see *il18* and a number of other scavenger receptors as DEGs. Given that KCs are derived from monocytes, we also expect that some of the findings between the 2 clusters are related. Possibly, increased activation in the monocytes and derived cells promotes a specific KC type as seen in the Figure 6 UMAP. When observing the downregulated gene expression, it is also possible there is a compensatory mechanism between cell types. The increased PGAM1 observed in Figure 7 with infected cells was suggestive of IL-27 producer influence on metabolism during infection. The KC population specifically displayed metabolic themes, an unsurprising finding given the metabolic role of the liver; however, this is supported by additional data from our lab demonstrating that IL-27 signaling is associated with low insulin levels and hypoglycemia.^37^

Neutrophil degranulation in response to infection was expected and likely contributes to inflammation and tissue damage, but IL-27 production from a cell engaged in such inflammatory activity was surprising. Disruption of protein synthesis, ribosome biogenesis, regulation of translation, and protein processing and folding could be a product of the degranulation event, tissue damage, and hypoxia, but directly suggest that endoplasmic reticulum stress is well characterized in neutrophils during sepsis.^59^  *Mmp9* and *Cxcr2*, classically associated with neutrophil activity and recruitment were increased in the neutrophil cluster.

The migratory cDC cluster was among the more interesting findings as these cells were the most significant expressors of IL-27 across cell types at the time analyzed postinfection. It is important to point out that while GFP is a reliable reporter of IL-27p28 gene activity, it does not necessary report current gene expression levels in a cell. The GFP needs to accumulate to accumulate for detection, and the kinetics of peak gene expression and peak protein production are not always identical. As such, at other time point not examined here, IL-27p28 transcripts are likely to be higher in other cell type clusters. Migratory cDCs demonstrated an intuitive expression pattern with *Ccr7* and *Ccl22* both being highly upregulated along with *Flt3*. Flt3 is an essential part of DC maturation, and *Ccr7* is highly expressed on mature DCs.^60^ This suggests DC recruitment followed by maturation and enhanced interaction with T cells promoted by CCL22.^61^ Although cDCs downregulated genes for phagocytosis and internalization of antigen, they increased expression of genes associated with antigen presentation and production of cytokines. While this activity and the regulation of Th17 cells may generally be considered beneficial during infection, the expression of *ccr7* and *ccl22* may suggest otherwise. The CCR7-CCL22 axis has been linked with hepatic T cell infiltration and activity during acute liver injury.^62^ Furthermore, CCL22 signaling through CCR4 has been shown to promote the formation of cell–cell contacts and interaction with regulatory T cells, as well as regulatory T cell–mediated suppressive activity during severe sepsis.^59^^,^^63^

To our knowledge, this is the first report to profile early-life IL-27 producers and their changes during neonatal infection. Together, these data provide key knowledge to aid in developing IL-27 as a therapeutic target. We were able to identify tissues suspected of IL-27 production and characterize the cell populations that make IL-27 during infection, which can be further explored to address specific downstream effects of blocking IL-27. In the wake of antimicrobial resistance, it is essential to continue to find new ways to combat life-threatening diseases, especially for one of our most vulnerable populations.

## Supplementary Material

vlaf026_Supplementary_Data

## Data Availability

The data supporting this body of work are available upon request by the corresponding author.
