# Supplementary material for: Interleukin-27–producing cells in gram-negative neonatal sepsis display diverse phenotypes and functions in the liver
Source: Immunohorizons. 2025 Jul 18;9(8):vlaf026. doi: 10.1093/immhor/vlaf026 (PMC12274645; doi:10.1093/immhor/vlaf026)
Supplement: vlaf026_Supplementary_Data [file vlaf026_supplementary_data.docx]

**Supplemental Materials**


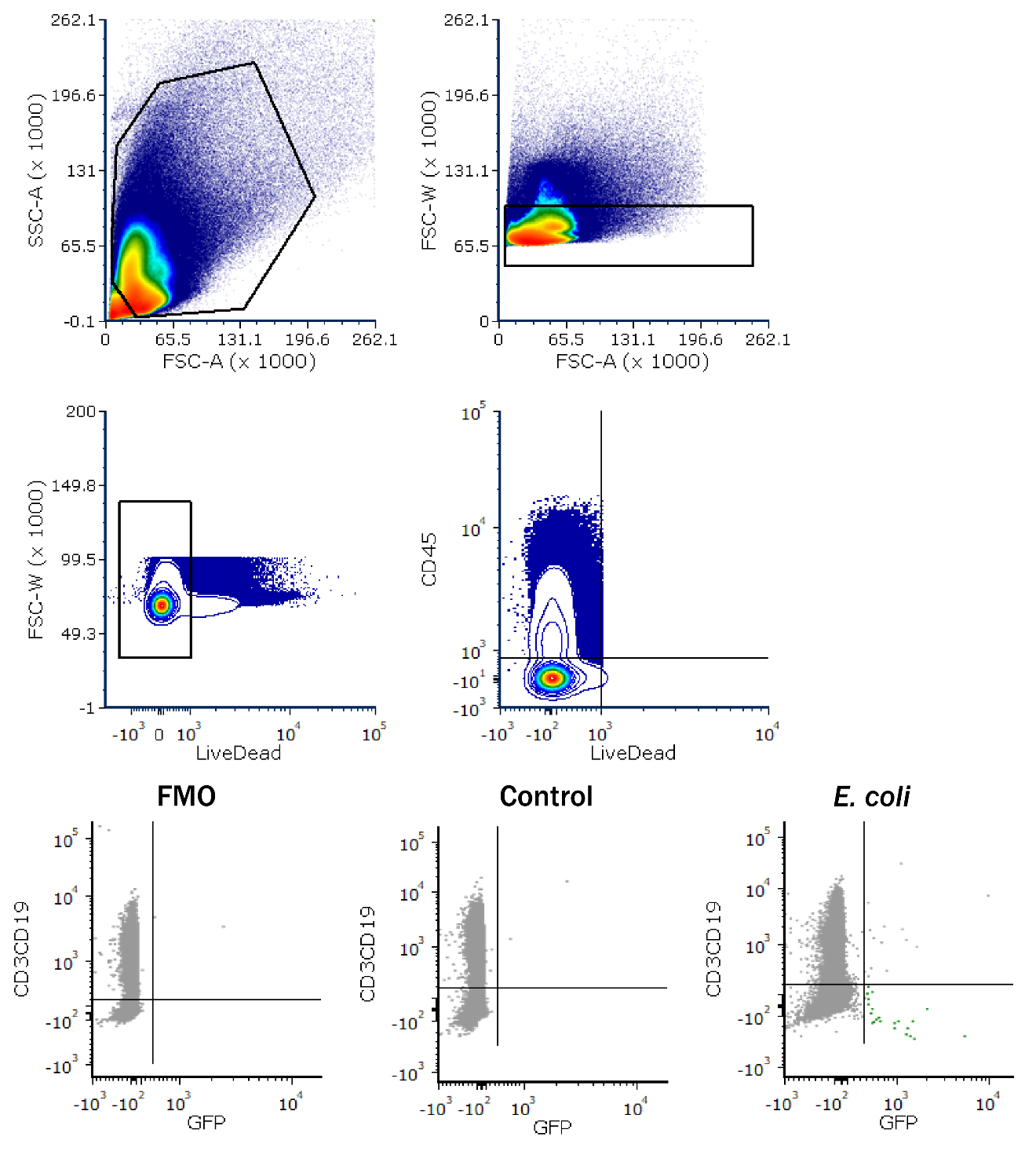


*Supplemental Figure 1. GFP^+^ cells are visible when viewing the live (FVS780^-^) immune (CD45^+^) non-lymphoid (CD3^-^/CD19^-^) population.* Representative plots were shown to demonstrate how the spleen was gated to determine GFP^+^ cells (green) and the resulting subpopulations.


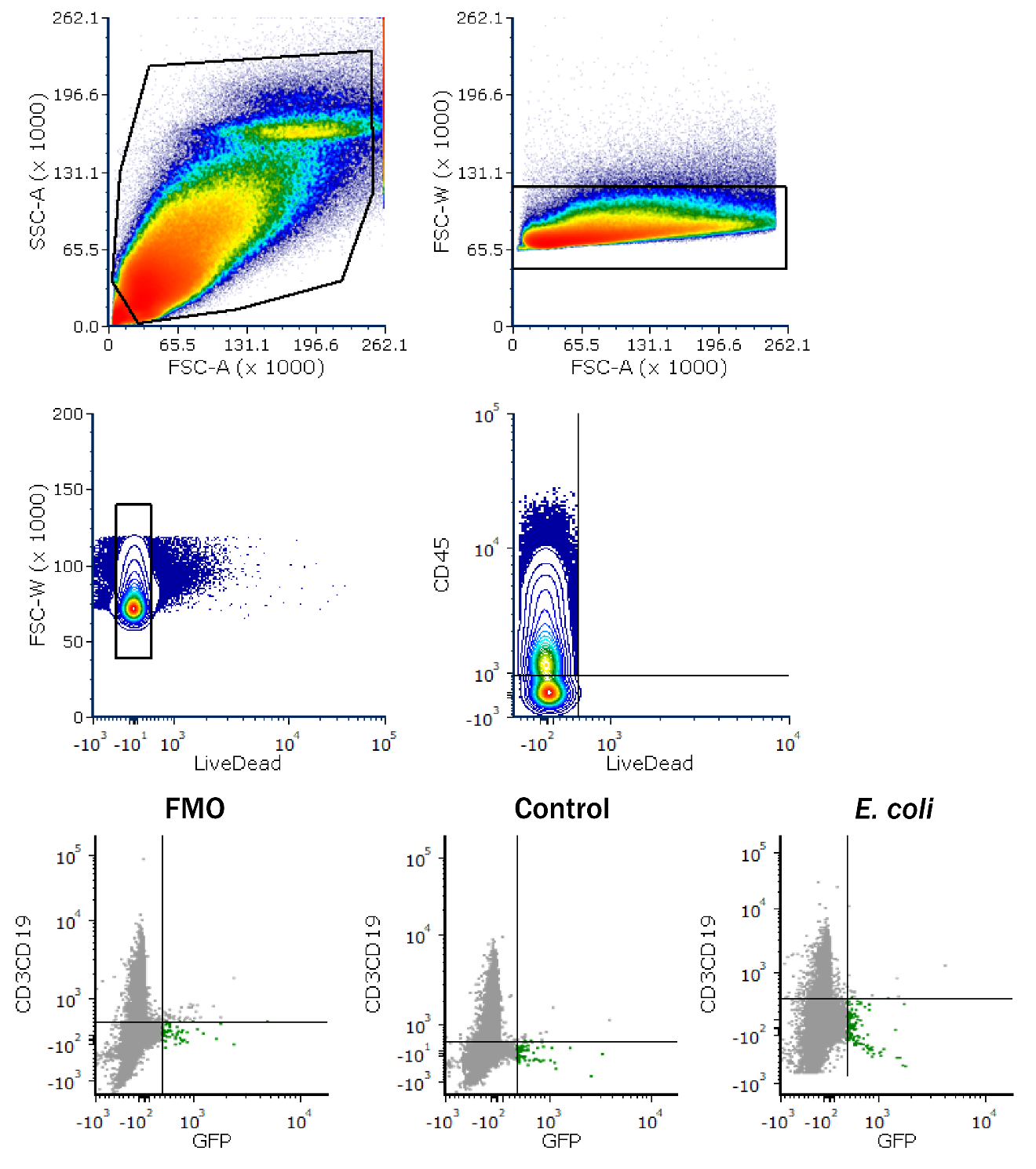


*Supplemental Figure 2. GFP^+^ cells are visible when viewing the live (FVS780^-^) immune (CD45^+^) non-lymphoid (CD3^-^/CD19^-^) population.* Representative plots were shown to demonstrate how the liver was gated to determine GFP^+^ cells (green) and the resulting subpopulations.


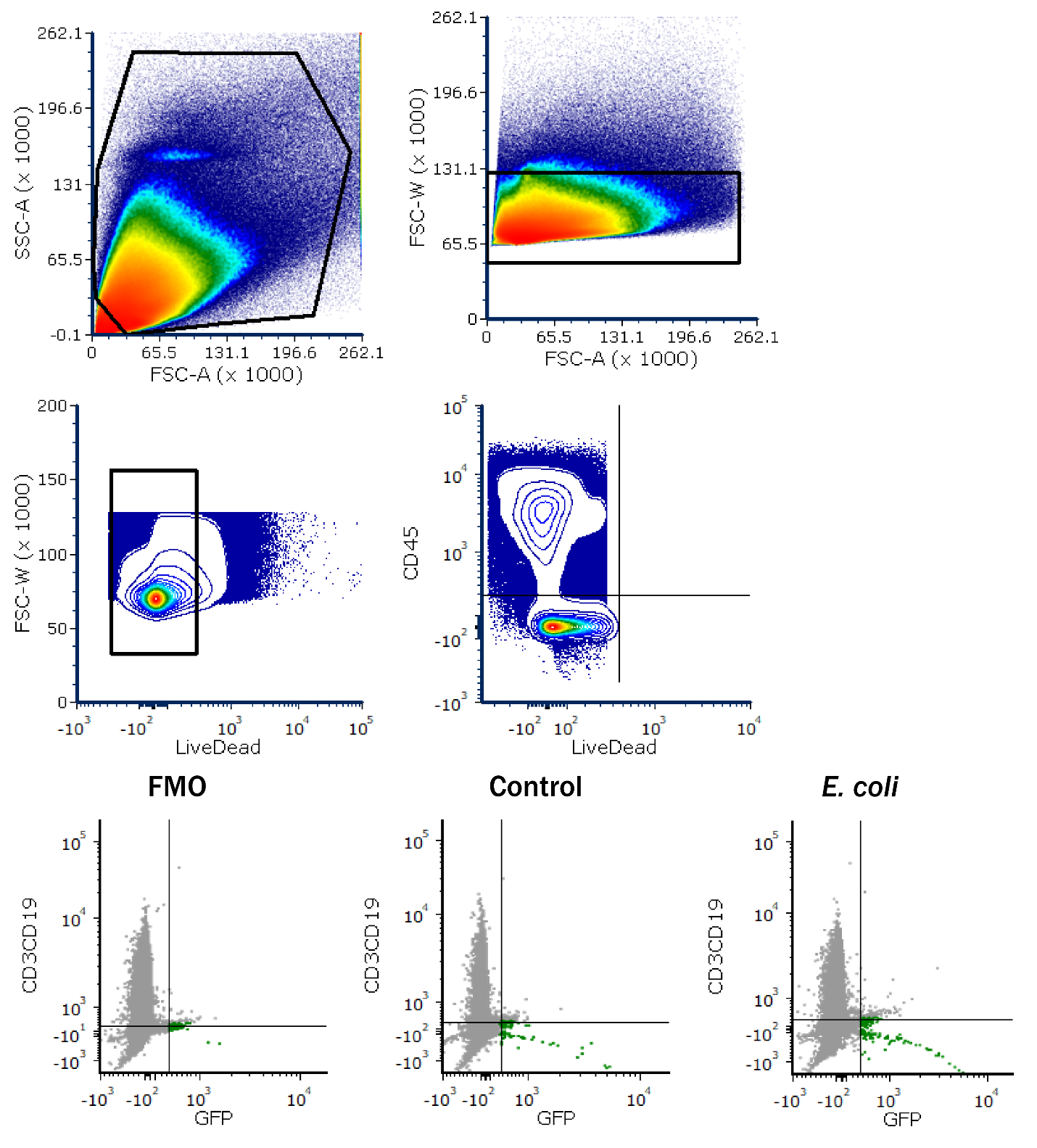


*Supplemental Figure 3. GFP^+^ cells are visible when viewing the live (FVS780^-^) immune (CD45^+^) non-lymphoid (CD3^-^/CD19^-^) population.* Representative plots were shown to demonstrate how the lung was gated to determine GFP^+^ cells (green) and the resulting subpopulations.


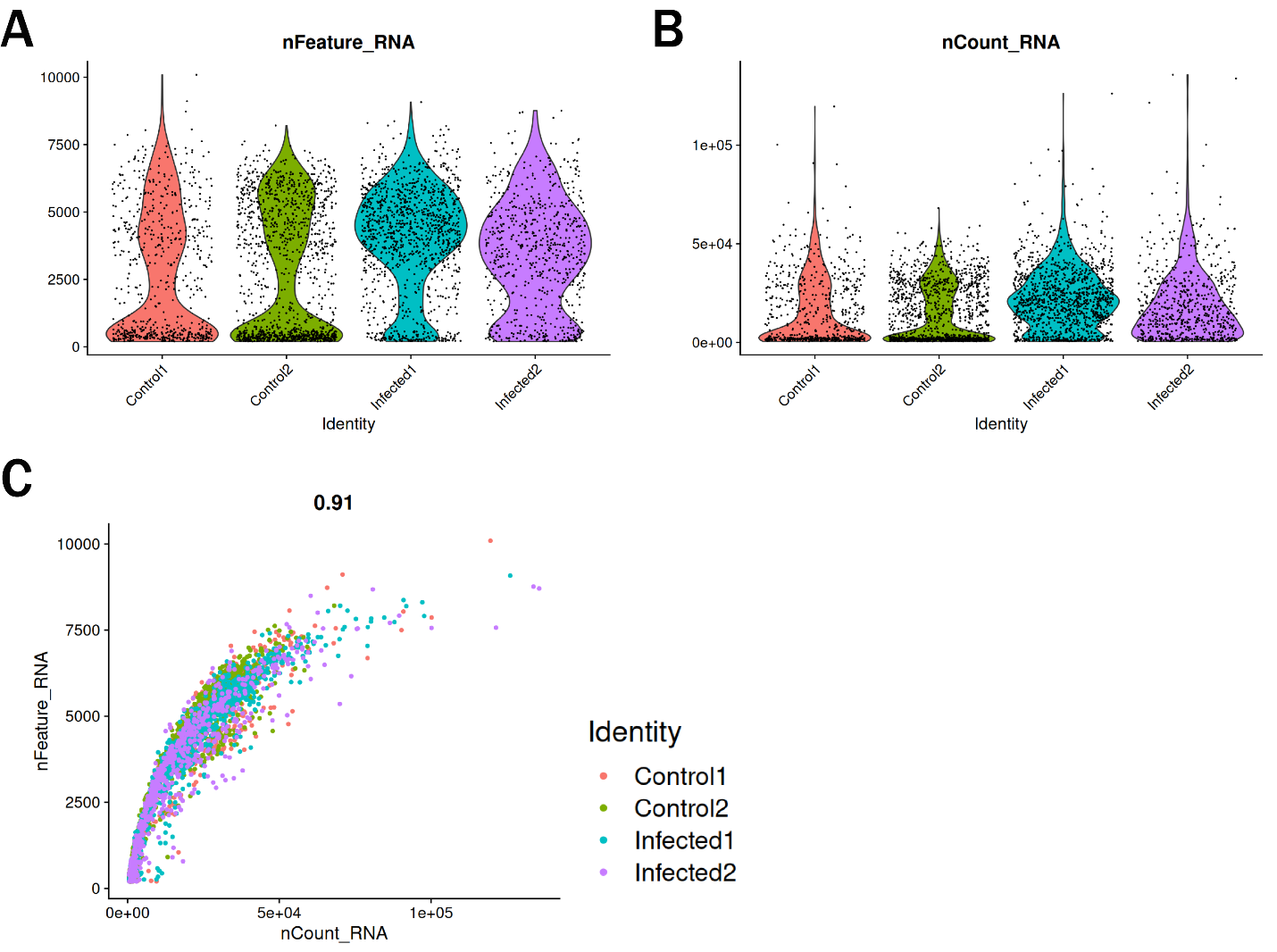


*Supplemental Figure 4. Quality control metrics indicated sound data quality.* (**A**) The number of detected genes per cell (nFeature) and (**B**) total unique molecular identifiers (UMI) counts per cell (nCount) were in acceptable ranges after filtering and were (**C**) correlated to one another (0.91).


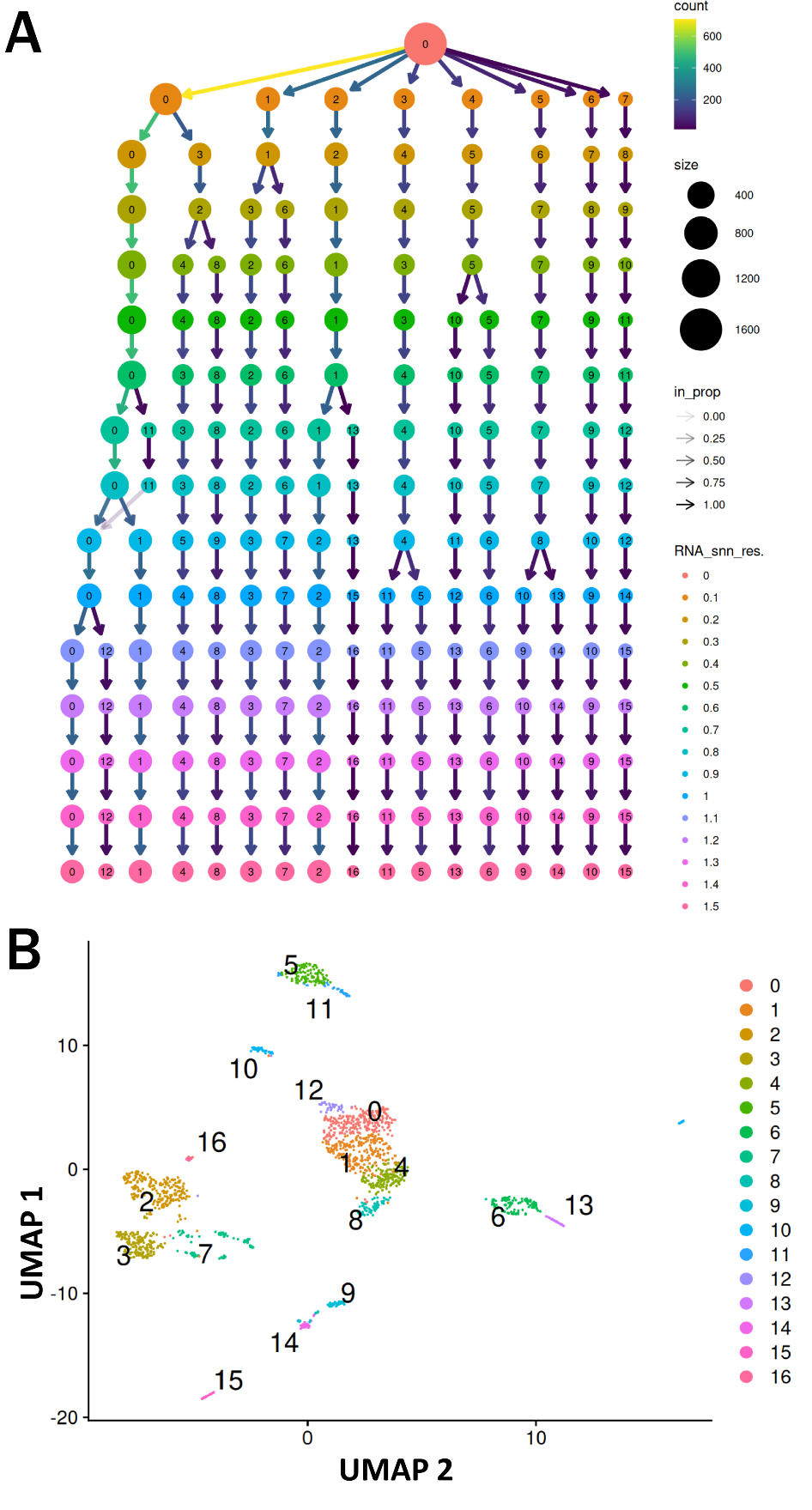


*Supplemental Figure 5. Scheme that clustered cells to the optimal resolution.* (**A**) The cells were clustered to the optimal resolution (16 clusters) according to the cluster tree. (**B**) Clustered cells were visualized by UMAP and labeled appropriately.
